# Bupropion hydro­bromide propanol hemisolvate

**DOI:** 10.1107/S1600536811037093

**Published:** 2011-09-30

**Authors:** Min Liu, Xiu-Rong Hu, Jian-Ming Gu, Gu-Ping Tang

**Affiliations:** a Chemistry Department, Zhejiang University, Hangzhou, Zhejiang 310028, People’s Republic of China; b Center of Analysis and Measurement, Zhejiang University, Hangzhou, Zhejiang 310028, People’s Republic of China

## Abstract

The title compound {systematic name: *N*-[1-(3-chloro­phen­yl)-1-oxopropan-2-yl]-*tert*-butanaminium bromide propanol hemisolvate}, C_13_H_19_ClNO^+^·Br^−^·0.5C_3_H_8_O, crystallizes with two independent bupropion hydro­bromide ion pairs and a solvent 1-propanol mol­ecule in the asymmetric unit. In both mol­ecules, the expected proton transfer from HBr to the amino group of the bupropion mol­ecule is observed, and intra- and inter­molecular N—H⋯Br hydrogen-bond inter­actions are formed. These inter­actions link the mol­ecules into hydrogen-bond dimers. The side chains of the two cations have slightly different orientations. The 1-propanol solvent mol­ecule is linked to a bromide ion by an O—H⋯Br hydrogen bond.

## Related literature

For applications of bupropion in the medicine field, see: Fryer *et al.* (1999 ▶); Stewart *et al.* (2001 ▶); Fang *et al.* (2000 ▶). For the related structures of an ethanol hemi-solvate bupropion derivative and bupropion hydro­chloride, see: Froimowitz *et al.* (1998 ▶); Maccaroni *et al.* (2009 ▶).
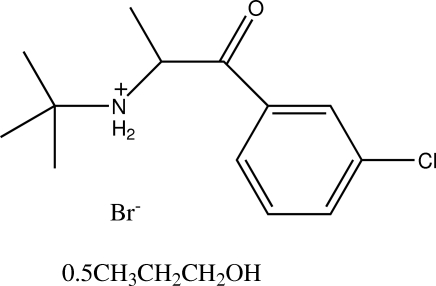


## Experimental

### 

#### Crystal data

C_13_H_19_ClNO^+^·Br^−^·0.5C_3_H_8_O
*M* *_r_* = 350.70
Triclinic, 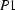

*a* = 7.8614 (4) Å
*b* = 9.4100 (6) Å
*c* = 11.8477 (7) Å
α = 85.783 (2)°
β = 78.159 (2)°
γ = 89.450 (2)°
*V* = 855.46 (9) Å^3^
*Z* = 2
Mo *K*α radiation
μ = 2.56 mm^−1^
*T* = 296 K
0.46 × 0.28 × 0.14 mm

#### Data collection

Rigaku R-AXIS RAPID diffractometer
Absorption correction: multi-scan (*ABSCOR*; Higashi, 1995 ▶) *T*_min_ = 0.361, *T*_max_ = 0.647
8456 measured reflections
6355 independent reflections
4179 reflections with *I* > 2σ(*I*)
*R*_int_ = 0.031

#### Refinement

*R*[*F* ^2^ > 2σ(*F* ^2^)] = 0.043
*wR*(*F* ^2^) = 0.138
*S* = 1.00
6355 reflections
354 parameters
77 restraints
H-atom parameters constrained
Δρ_max_ = 0.74 e Å^−3^
Δρ_min_ = −0.97 e Å^−3^
Absolute structure: Flack (1983 ▶), 2490 Friedel pairs
Flack parameter: 0.34 (3)

### 

Data collection: *PROCESS-AUTO* (Rigaku, 2006 ▶); cell refinement: *PROCESS-AUTO*; data reduction: *CrystalStructure* (Rigaku, 2007 ▶); program(s) used to solve structure: *SHELXS97* (Sheldrick, 2008 ▶); program(s) used to refine structure: *SHELXL97* (Sheldrick, 2008 ▶); molecular graphics: *ORTEP-3 for Windows* (Farrugia, 1997 ▶); software used to prepare material for publication: *WinGX* (Farrugia, 1999 ▶).

## Supplementary Material

Crystal structure: contains datablock(s) global, I. DOI: 10.1107/S1600536811037093/bx2372sup1.cif

Structure factors: contains datablock(s) I. DOI: 10.1107/S1600536811037093/bx2372Isup2.hkl

Supplementary material file. DOI: 10.1107/S1600536811037093/bx2372Isup3.cml

Additional supplementary materials:  crystallographic information; 3D view; checkCIF report

## Figures and Tables

**Table 1:** Hydrogen-bond geometry (Å, °)

*D*—H⋯*A*	*D*—H	H⋯*A*	*D*⋯*A*	*D*—H⋯*A*
N1*A*—H1*A*1⋯Br1*A*	0.90	2.46	3.353 (9)	174
N1*A*—H1*A*2⋯Br1*B*^i^	0.90	2.60	3.410 (9)	150
N1*B*—H1*B*1⋯Br1*B*^ii^	0.90	2.46	3.362 (9)	175
N1*B*—H1*B*2⋯Br1*A*^iii^	0.90	2.58	3.383 (9)	149
O21—H21⋯Br1*A*	0.82	2.73	3.487 (10)	153

Symmetry codes: (i) 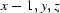; (ii) 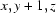; (iii) 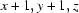.

**Fig. 1.**
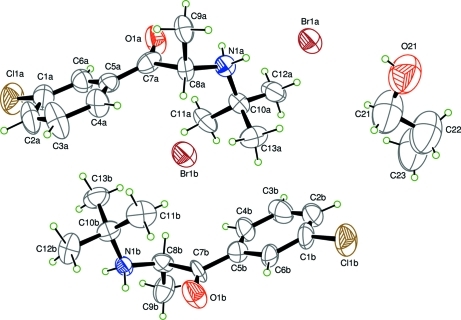
Molecular structure of the title compound (1) showing atom-labelling scheme and displacement ellipsoids at 40% probability level. H atoms are shown as small circles of arbitrary radii.

## supplementary crystallographic information

### Comment

The title compound, bupropion hydrobromide, 1-(3-chlorophenyl)-2-[(1,1- dimethylethyl)amino]-1-propanone hydrobromide, belongs to the class of antidepressants known as aminoketones and it is known also with the drug name Aplenzin. It is a second generation antidepressant approved in US and in some European countries, its mechanism of action, both as an antidepressant and as an aids to smoking cessation, is thought to involve nicotinic acetylcholine receptors that are linked to dopamine and norepinephrine release (Fryer *et al.* 1999 & Stewart *et al.*, 2001). Pure bupropion enantiomers were successfully synthesized but they give rise to a rapid racemization (Fang *et al.*, 2000). In literature, crystal structure of an ethanol hemi-solvate bupropion derivative and bupropion hydrochloride, obtained from single-crystal X-ray analysis and powder diffraction, were reported (Froimowitz *et al.*, 1998 & Maccaroni *et al.*, 2009). Here, we reported crystal structure of bupropion hydrobromide propanol solvate. The asymmetric unit consists of two bupropion cations, two bromide anions and one 1-propanol molecule (Fig.1). Expected proton transfer from HBr to amino group of bupropion is observed, intramolecular and intermolecular hydrogen bond interactions are formed (Table 1).These interactions result in hydrogen-bond dimers in the two polymorphic forms, in which two Br^-^ ions bridge the NH_2_—NH_2_ contact (above 4.2 Å), similar to that of BUP hydrochloride (Maccaroni *et al.*, 2009). Solvent molecule 1-propanol is linked to bupropion hydrobromide by intramolecular hydrogen bond O21—H21···Br1A. The side chains of the two molecules have slightly different orientations, as seen by the torsion angles of C6—C5—C7—C8, C5—C7—C8—N1, C7—C8—N1—C10 and O1—C7—C5—C6. Carbonyl groups in the two molecules are not coplanar with phenyl ring plane, atom O1A and O1B deviated from the least-squares plane of phenyl ring (C1A/C6A and C1B/C6B) 0.238Å and 0.139 Å, respectively.

### Experimental

The crude product is supplied by Zhejiang Apeloa Pharmaceutical Co.,LTD. It was recrystallized from 1-propanol solution, giving colorless crystals of (1) suitable for X-ray diffraction.

### Refinement

The residual electron density  to indicate the presence of a possible H atom on the atoms N1A and N1B, showing that a proton transfer from HBr to amino group of bupropion molecule. These H atoms were placed in calculated positions with N—H = 0.90Å and refined as riding with *U*_iso_(H) = 1.2*U*_eq_(N). All other H atoms were placed in calculated positions with C—H = 0.93–0.99Å and included in the refinement in riding model, with *U*_iso_(H) = 1.2*U*_eq_ or 1.5*U*_eq_(carrier atom). Temperature factor of atom O21, C21, C22 and C23 from solvent molecule were restrained with effective standard deviations so that their *U^ij^* components approximate to isotropic behavior; however the corresponding isotropic U is free to vary.

### Crystal data

**Table:**

C_13_H_19_ClNO^+^·Br^−^·0.5C_3_H_8_O	*Z* = 2
*M**_r_* = 350.70	*F*(000) = 362
Triclinic, *P*1	*D*_x_ = 1.361 Mg m^−^^3^
Hall symbol: P 1	Mo *K*α radiation, λ = 0.71073 Å
*a* = 7.8614 (4) Å	Cell parameters from 5965 reflections
*b* = 9.4100 (6) Å	θ = 3.4–27.4°
*c* = 11.8477 (7) Å	µ = 2.56 mm^−^^1^
α = 85.783 (2)°	*T* = 296 K
β = 78.159 (2)°	Chunk, colorless
γ = 89.450 (2)°	0.46 × 0.28 × 0.14 mm
*V* = 855.46 (9) Å^3^	

### Data collection

**Table:**

Rigaku R-AXIS RAPID diffractometer	6355 independent reflections
Radiation source: rolling anode	4179 reflections with *I* > 2σ(*I*)
graphite	*R*_int_ = 0.031
Detector resolution: 10.00 pixels mm^-1^	θ_max_ = 27.5°, θ_min_ = 3.4°
ω scans	*h* = −10→8
Absorption correction: multi-scan (*ABSCOR*; Higashi, 1995)	*k* = −12→12
*T*_min_ = 0.361, *T*_max_ = 0.647	*l* = −15→15
8456 measured reflections	

### Refinement

**Table:**

Refinement on *F*^2^	Hydrogen site location: inferred from neighbouring sites
Least-squares matrix: full	H-atom parameters constrained
*R*[*F*^2^ > 2σ(*F*^2^)] = 0.043	*w* = 1/[σ^2^(*F*_o_^2^) + (0.0295*P*)^2^ + 2.750*P*] where *P* = (*F*_o_^2^ + 2*F*_c_^2^)/3
*wR*(*F*^2^) = 0.138	(Δ/σ)_max_ = 0.002
*S* = 1.00	Δρ_max_ = 0.74 e Å^−^^3^
6355 reflections	Δρ_min_ = −0.97 e Å^−^^3^
354 parameters	Extinction correction: *SHELXL*, Fc^*^=kFc[1+0.001xFc^2^λ^3^/sin(2θ)]^-1/4^
77 restraints	Extinction coefficient: 0.038 (2)
Primary atom site location: structure-invariant direct methods	Absolute structure: Flack (1983), 2490 Friedel pairs
Secondary atom site location: difference Fourier map	Flack parameter: 0.34 (3)

### Special details

**Table:**

Geometry. All e.s.d.'s (except the e.s.d. in the dihedral angle between two l.s. planes) are estimated using the full covariance matrix. The cell e.s.d.'s are taken into account individually in the estimation of e.s.d.'s in distances, angles and torsion angles; correlations between e.s.d.'s in cell parameters are only used when they are defined by crystal symmetry. An approximate (isotropic) treatment of cell e.s.d.'s is used for estimating e.s.d.'s involving l.s. planes.
Refinement. Refinement of *F*^2^ against ALL reflections. The weighted *R*-factor *wR* and goodness of fit *S* are based on *F*^2^, conventional *R*-factors *R* are based on *F*, with *F* set to zero for negative *F*^2^. The threshold expression of *F*^2^ > σ(*F*^2^) is used only for calculating *R*-factors(gt) *etc*. and is not relevant to the choice of reflections for refinement. *R*-factors based on *F*^2^ are statistically about twice as large as those based on *F*, and *R*- factors based on ALL data will be even larger.

### Fractional atomic coordinates and isotropic or equivalent isotropic displacement parameters (Å^2^)

**Table:**

	*x*	*y*	*z*	*U*_iso_*/*U*_eq_	
Br1A	0.2817 (5)	0.0940 (4)	0.4424 (3)	0.0574 (4)	
Br1B	0.8907 (5)	0.3667 (4)	0.2779 (3)	0.0577 (4)	
Cl1A	0.5787 (10)	1.0426 (6)	−0.1054 (6)	0.096 (2)	
Cl1B	0.5901 (10)	0.4189 (6)	0.8270 (6)	0.099 (2)	
N1A	0.2948 (10)	0.4307 (9)	0.3266 (6)	0.044 (3)	
H1A1	0.2916	0.3381	0.3520	0.053*	
H1A2	0.1945	0.4498	0.3032	0.053*	
O1A	0.2393 (18)	0.6111 (14)	0.1487 (11)	0.073 (4)	
C5B	0.6396 (10)	0.7943 (10)	0.6451 (12)	0.049 (3)	
N1B	0.8848 (10)	1.0271 (9)	0.3887 (6)	0.041 (3)	
H1B1	0.8891	1.1197	0.3632	0.049*	
H1B2	0.9870	1.0066	0.4091	0.049*	
C7A	0.398 (2)	0.5823 (16)	0.1472 (13)	0.055 (4)	
C8A	0.4388 (13)	0.4504 (9)	0.2238 (8)	0.045 (3)	
H8A	0.5518	0.4593	0.2455	0.054*	
O1B	0.9229 (17)	0.8444 (14)	0.5699 (13)	0.080 (4)	
C10B	0.8749 (8)	0.9430 (6)	0.2890 (6)	0.050 (4)	
C6B	0.670 (2)	0.6656 (11)	0.7018 (12)	0.053 (4)	
H6B	0.7841	0.6344	0.6958	0.063*	
C13A	0.4498 (12)	0.4659 (13)	0.4839 (11)	0.062 (4)	
H13A	0.5548	0.4633	0.4260	0.092*	
H13B	0.4246	0.3723	0.5214	0.092*	
H13C	0.4645	0.5308	0.5401	0.092*	
C1A	0.6250 (10)	0.8758 (9)	−0.0469 (12)	0.064 (5)	
C10A	0.2985 (8)	0.5161 (6)	0.4266 (6)	0.051 (4)	
C8B	0.7476 (14)	1.0115 (9)	0.4957 (8)	0.049 (3)	
H8B	0.6371	0.9965	0.4721	0.059*	
C6A	0.500 (2)	0.7921 (12)	0.0245 (13)	0.059 (4)	
H6A	0.3846	0.8209	0.0386	0.071*	
C4B	0.4675 (9)	0.8365 (11)	0.6632 (12)	0.069 (4)	
H4B	0.4410	0.9225	0.6268	0.083*	
C4A	0.7179 (8)	0.6209 (11)	0.0595 (12)	0.058 (4)	
H4A	0.7482	0.5365	0.0963	0.070*	
C11B	0.846 (2)	0.7831 (7)	0.3252 (16)	0.071 (5)	
H11A	0.8494	0.7317	0.2577	0.106*	
H11B	0.7354	0.7690	0.3766	0.106*	
H11C	0.9365	0.7489	0.3638	0.106*	
C5A	0.5463 (9)	0.6643 (11)	0.0754 (12)	0.048 (3)	
C11A	0.321 (2)	0.6762 (8)	0.3895 (15)	0.070 (5)	
H11D	0.4355	0.6937	0.3436	0.105*	
H11E	0.3054	0.7295	0.4569	0.105*	
H11F	0.2361	0.7054	0.3448	0.105*	
C12B	1.0499 (12)	0.9757 (14)	0.2054 (10)	0.061 (4)	
H12A	1.0684	1.0769	0.1948	0.091*	
H12B	1.0482	0.9388	0.1322	0.091*	
H12C	1.1422	0.9317	0.2370	0.091*	
C12A	0.1247 (13)	0.4992 (16)	0.5144 (11)	0.074 (5)	
H12D	0.1256	0.5583	0.5770	0.112*	
H12E	0.1089	0.4015	0.5440	0.112*	
H12F	0.0310	0.5274	0.4770	0.112*	
C13B	0.7260 (13)	1.0021 (17)	0.2329 (11)	0.071 (5)	
H13D	0.7401	1.1032	0.2168	0.107*	
H13E	0.6168	0.9820	0.2849	0.107*	
H13F	0.7284	0.9578	0.1622	0.107*	
C2B	0.3677 (10)	0.6293 (11)	0.7882 (13)	0.070 (5)	
H2B	0.2803	0.5765	0.8381	0.084*	
C7B	0.7805 (18)	0.8790 (15)	0.5713 (13)	0.049 (4)	
C9A	0.433 (2)	0.3227 (11)	0.1505 (11)	0.057 (4)	
H9A1	0.4510	0.2362	0.1946	0.085*	
H9A2	0.5222	0.3333	0.0818	0.085*	
H9A3	0.3214	0.3189	0.1295	0.085*	
C1B	0.5383 (12)	0.5830 (9)	0.7665 (12)	0.067 (5)	
C2A	0.7974 (10)	0.8344 (13)	−0.0634 (13)	0.082 (6)	
H2A	0.8830	0.8932	−0.1090	0.098*	
C3B	0.3327 (15)	0.7575 (12)	0.7325 (11)	0.074 (5)	
H3B	0.2191	0.7904	0.7415	0.088*	
C9B	0.728 (3)	1.1461 (12)	0.5637 (12)	0.072 (5)	
H9B1	0.6318	1.1337	0.6283	0.108*	
H9B2	0.7072	1.2269	0.5140	0.108*	
H9B3	0.8325	1.1615	0.5911	0.108*	
C3A	0.8433 (17)	0.7064 (12)	−0.0127 (14)	0.089 (6)	
H3A	0.9588	0.6777	−0.0271	0.106*	
O21	0.0850 (16)	0.0562 (11)	0.7350 (9)	0.140 (3)	
H21	0.0956	0.0584	0.6646	0.211*	
C23	0.016 (3)	0.3261 (16)	0.9379 (14)	0.167 (5)	
H23A	0.0930	0.3666	0.9807	0.250*	
H23B	−0.0973	0.3139	0.9861	0.250*	
H23C	0.0098	0.3888	0.8713	0.250*	
C21	0.142 (3)	0.1909 (14)	0.7667 (10)	0.152 (4)	
H21A	0.0864	0.2703	0.7323	0.183*	
H21B	0.2673	0.2019	0.7426	0.183*	
C22	0.086 (3)	0.1810 (15)	0.8990 (10)	0.158 (4)	
H22A	0.1850	0.1546	0.9336	0.190*	
H22B	−0.0030	0.1086	0.9240	0.190*	

### Atomic displacement parameters (Å^2^)

**Table:**

	*U*^11^	*U*^22^	*U*^33^	*U*^12^	*U*^13^	*U*^23^
Br1A	0.0472 (9)	0.0500 (9)	0.0750 (11)	−0.0012 (7)	−0.0172 (8)	0.0093 (8)
Br1B	0.0486 (9)	0.0505 (9)	0.0743 (11)	−0.0007 (7)	−0.0185 (8)	0.0109 (8)
Cl1A	0.138 (6)	0.060 (3)	0.082 (4)	−0.010 (3)	−0.018 (4)	0.024 (3)
Cl1B	0.150 (7)	0.058 (3)	0.088 (4)	−0.022 (3)	−0.031 (4)	0.027 (3)
N1A	0.039 (6)	0.041 (6)	0.053 (7)	0.006 (5)	−0.013 (5)	0.003 (5)
O1A	0.058 (8)	0.069 (7)	0.084 (8)	−0.004 (6)	−0.011 (6)	0.031 (6)
C5B	0.047 (7)	0.047 (8)	0.051 (8)	0.005 (6)	−0.010 (6)	−0.002 (6)
N1B	0.033 (6)	0.042 (6)	0.046 (6)	−0.004 (5)	−0.006 (5)	0.006 (5)
C7A	0.059 (9)	0.060 (9)	0.053 (8)	0.017 (7)	−0.031 (7)	0.000 (7)
C8A	0.039 (6)	0.044 (7)	0.046 (7)	0.002 (5)	0.001 (5)	0.012 (6)
O1B	0.041 (7)	0.075 (8)	0.120 (10)	−0.003 (6)	−0.021 (7)	0.038 (8)
C10B	0.050 (9)	0.051 (8)	0.052 (8)	0.010 (7)	−0.017 (7)	−0.011 (7)
C6B	0.073 (11)	0.039 (7)	0.046 (7)	−0.007 (7)	−0.017 (7)	0.013 (6)
C13A	0.077 (11)	0.046 (7)	0.073 (10)	0.017 (7)	−0.039 (9)	−0.014 (7)
C1A	0.080 (13)	0.056 (9)	0.052 (9)	−0.006 (9)	−0.004 (9)	−0.003 (8)
C10A	0.043 (9)	0.047 (8)	0.059 (8)	−0.008 (7)	−0.005 (7)	−0.002 (7)
C8B	0.035 (6)	0.048 (7)	0.066 (9)	0.004 (5)	−0.018 (6)	0.002 (6)
C6A	0.056 (10)	0.060 (9)	0.057 (8)	−0.005 (8)	−0.003 (7)	−0.005 (7)
C4B	0.096 (11)	0.045 (7)	0.066 (9)	−0.025 (7)	−0.016 (8)	0.010 (6)
C4A	0.029 (5)	0.070 (8)	0.066 (8)	0.016 (5)	0.007 (5)	0.000 (7)
C11B	0.078 (12)	0.046 (8)	0.095 (12)	0.025 (7)	−0.029 (9)	−0.014 (8)
C5A	0.050 (7)	0.047 (7)	0.049 (8)	−0.004 (6)	−0.021 (6)	0.003 (6)
C11A	0.066 (10)	0.047 (8)	0.091 (11)	−0.021 (7)	−0.002 (9)	−0.006 (8)
C12B	0.059 (8)	0.067 (7)	0.045 (6)	0.034 (6)	0.017 (6)	−0.011 (6)
C12A	0.070 (9)	0.066 (8)	0.093 (10)	−0.024 (7)	−0.034 (8)	0.008 (7)
C13B	0.048 (9)	0.112 (13)	0.058 (9)	0.005 (8)	−0.019 (7)	−0.015 (9)
C2B	0.089 (13)	0.061 (10)	0.059 (9)	−0.024 (9)	−0.011 (8)	−0.001 (8)
C7B	0.032 (7)	0.046 (7)	0.061 (8)	−0.018 (6)	0.004 (6)	0.014 (6)
C9A	0.064 (9)	0.040 (6)	0.059 (8)	−0.002 (6)	0.000 (7)	0.011 (6)
C1B	0.109 (16)	0.043 (8)	0.045 (8)	−0.021 (9)	−0.011 (9)	0.013 (7)
C2A	0.090 (15)	0.069 (11)	0.067 (10)	−0.020 (10)	0.030 (9)	0.001 (9)
C3B	0.049 (9)	0.091 (13)	0.074 (11)	−0.011 (9)	0.008 (8)	−0.019 (9)
C9B	0.097 (14)	0.066 (9)	0.048 (7)	0.014 (9)	−0.004 (8)	−0.007 (7)
C3A	0.075 (13)	0.062 (10)	0.107 (14)	−0.004 (9)	0.028 (10)	0.010 (10)
O21	0.176 (9)	0.144 (8)	0.092 (6)	0.003 (7)	−0.009 (6)	−0.006 (5)
C23	0.241 (12)	0.138 (11)	0.104 (8)	0.023 (11)	0.000 (9)	0.007 (8)
C21	0.218 (10)	0.127 (9)	0.100 (7)	0.019 (9)	−0.009 (7)	0.006 (6)
C22	0.234 (10)	0.133 (10)	0.097 (7)	0.018 (9)	−0.013 (8)	−0.005 (6)

### Geometric parameters (Å, °)

**Table:**

Cl1A—C1A	1.735 (11)	C4A—H4A	0.9300
Cl1B—C1B	1.735 (11)	C11B—H11A	0.9601
N1A—C8A	1.485 (12)	C11B—H11B	0.9601
N1A—C10A	1.485 (10)	C11B—H11C	0.9601
N1A—H1A1	0.9000	C11A—H11D	0.9601
N1A—H1A2	0.9000	C11A—H11E	0.9601
O1A—C7A	1.271 (19)	C11A—H11F	0.9601
C5B—C4B	1.385 (12)	C12B—H12A	0.9600
C5B—C6B	1.384 (16)	C12B—H12B	0.9600
C5B—C7B	1.464 (15)	C12B—H12C	0.9600
N1B—C10B	1.485 (10)	C12A—H12D	0.9600
N1B—C8B	1.485 (12)	C12A—H12E	0.9600
N1B—H1B1	0.9000	C12A—H12F	0.9600
N1B—H1B2	0.9000	C13B—H13D	0.9600
C7A—C5A	1.481 (19)	C13B—H13E	0.9600
C7A—C8A	1.553 (17)	C13B—H13F	0.9600
C8A—C9A	1.540 (14)	C2B—C3B	1.385 (16)
C8A—H8A	0.9800	C2B—C1B	1.385 (13)
O1B—C7B	1.160 (18)	C2B—H2B	0.9300
C10B—C13B	1.541 (13)	C9A—H9A1	0.9600
C10B—C11B	1.540 (10)	C9A—H9A2	0.9600
C10B—C12B	1.540 (12)	C9A—H9A3	0.9600
C6B—C1B	1.367 (17)	C2A—C3A	1.385 (17)
C6B—H6B	0.9300	C2A—H2A	0.9300
C13A—C10A	1.540 (12)	C3B—H3B	0.9300
C13A—H13A	0.9600	C9B—H9B1	0.9600
C13A—H13B	0.9600	C9B—H9B2	0.9600
C13A—H13C	0.9600	C9B—H9B3	0.9600
C1A—C6A	1.370 (16)	C3A—H3A	0.9300
C1A—C2A	1.385 (12)	O21—C21	1.449 (18)
C10A—C11A	1.540 (11)	O21—H21	0.8200
C10A—C12A	1.540 (12)	C23—C22	1.530 (19)
C8B—C7B	1.532 (17)	C23—H23A	0.9600
C8B—C9B	1.540 (15)	C23—H23B	0.9600
C8B—H8B	0.9800	C23—H23C	0.9600
C6A—C5A	1.385 (17)	C21—C22	1.535 (17)
C6A—H6A	0.9300	C21—H21A	0.9700
C4B—C3B	1.385 (16)	C21—H21B	0.9700
C4B—H4B	0.9300	C22—H22A	0.9700
C4A—C3A	1.385 (17)	C22—H22B	0.9700
C4A—C5A	1.385 (11)		
			
C8A—N1A—C10A	118.2 (8)	C10A—C11A—H11E	109.5
C8A—N1A—H1A1	107.8	H11D—C11A—H11E	109.5
C10A—N1A—H1A1	107.8	C10A—C11A—H11F	109.5
C8A—N1A—H1A2	107.8	H11D—C11A—H11F	109.5
C10A—N1A—H1A2	107.8	H11E—C11A—H11F	109.5
H1A1—N1A—H1A2	107.1	C10B—C12B—H12A	109.5
C4B—C5B—C6B	115.4 (11)	C10B—C12B—H12B	109.5
C4B—C5B—C7B	122.8 (10)	H12A—C12B—H12B	109.5
C6B—C5B—C7B	121.9 (10)	C10B—C12B—H12C	109.5
C10B—N1B—C8B	120.2 (8)	H12A—C12B—H12C	109.5
C10B—N1B—H1B1	107.3	H12B—C12B—H12C	109.5
C8B—N1B—H1B1	107.3	C10A—C12A—H12D	109.5
C10B—N1B—H1B2	107.3	C10A—C12A—H12E	109.5
C8B—N1B—H1B2	107.3	H12D—C12A—H12E	109.5
H1B1—N1B—H1B2	106.9	C10A—C12A—H12F	109.5
O1A—C7A—C5A	124.3 (12)	H12D—C12A—H12F	109.5
O1A—C7A—C8A	117.8 (13)	H12E—C12A—H12F	109.5
C5A—C7A—C8A	117.9 (11)	C10B—C13B—H13D	109.5
N1A—C8A—C9A	107.1 (9)	C10B—C13B—H13E	109.5
N1A—C8A—C7A	108.5 (10)	H13D—C13B—H13E	109.5
C9A—C8A—C7A	105.0 (10)	C10B—C13B—H13F	109.4
N1A—C8A—H8A	112.0	H13D—C13B—H13F	109.5
C9A—C8A—H8A	111.9	H13E—C13B—H13F	109.5
C7A—C8A—H8A	112.0	C3B—C2B—C1B	117.2 (10)
N1B—C10B—C13B	108.6 (8)	C3B—C2B—H2B	121.4
N1B—C10B—C11B	112.0 (9)	C1B—C2B—H2B	121.4
C13B—C10B—C11B	110.3 (10)	O1B—C7B—C5B	118.7 (13)
N1B—C10B—C12B	103.2 (7)	O1B—C7B—C8B	118.6 (11)
C13B—C10B—C12B	109.3 (9)	C5B—C7B—C8B	122.7 (11)
C11B—C10B—C12B	113.1 (10)	C8A—C9A—H9A1	109.5
C1B—C6B—C5B	122.1 (12)	C8A—C9A—H9A2	109.5
C1B—C6B—H6B	119.0	H9A1—C9A—H9A2	109.5
C5B—C6B—H6B	119.0	C8A—C9A—H9A3	109.5
C10A—C13A—H13A	109.5	H9A1—C9A—H9A3	109.5
C10A—C13A—H13B	109.5	H9A2—C9A—H9A3	109.5
H13A—C13A—H13B	109.5	C6B—C1B—C2B	121.9 (8)
C10A—C13A—H13C	109.5	C6B—C1B—Cl1B	118.3 (8)
H13A—C13A—H13C	109.5	C2B—C1B—Cl1B	119.8 (8)
H13B—C13A—H13C	109.5	C1A—C2A—C3A	120.6 (11)
C6A—C1A—C2A	119.5 (8)	C1A—C2A—H2A	119.7
C6A—C1A—Cl1A	122.1 (7)	C3A—C2A—H2A	119.7
C2A—C1A—Cl1A	118.0 (8)	C2B—C3B—C4B	119.7 (12)
N1A—C10A—C11A	111.6 (9)	C2B—C3B—H3B	120.1
N1A—C10A—C12A	109.8 (7)	C4B—C3B—H3B	120.1
C11A—C10A—C12A	106.6 (9)	C8B—C9B—H9B1	109.5
N1A—C10A—C13A	109.4 (8)	C8B—C9B—H9B2	109.5
C11A—C10A—C13A	109.1 (10)	H9B1—C9B—H9B2	109.5
C12A—C10A—C13A	110.3 (9)	C8B—C9B—H9B3	109.5
N1B—C8B—C7B	110.4 (9)	H9B1—C9B—H9B3	109.5
N1B—C8B—C9B	112.6 (9)	H9B2—C9B—H9B3	109.5
C7B—C8B—C9B	111.6 (11)	C4A—C3A—C2A	120.3 (13)
N1B—C8B—H8B	107.3	C4A—C3A—H3A	119.9
C7B—C8B—H8B	107.3	C2A—C3A—H3A	119.9
C9B—C8B—H8B	107.3	C21—O21—H21	109.5
C1A—C6A—C5A	119.7 (12)	C22—C23—H23A	109.5
C1A—C6A—H6A	120.1	C22—C23—H23B	109.5
C5A—C6A—H6A	120.1	H23A—C23—H23B	109.5
C5B—C4B—C3B	123.5 (11)	C22—C23—H23C	109.5
C5B—C4B—H4B	118.3	H23A—C23—H23C	109.5
C3B—C4B—H4B	118.3	H23B—C23—H23C	109.5
C3A—C4A—C5A	118.3 (12)	O21—C21—C22	103.6 (11)
C3A—C4A—H4A	120.9	O21—C21—H21A	111.1
C5A—C4A—H4A	120.9	C22—C21—H21A	111.0
C10B—C11B—H11A	109.5	O21—C21—H21B	111.0
C10B—C11B—H11B	109.5	C22—C21—H21B	111.0
H11A—C11B—H11B	109.5	H21A—C21—H21B	109.0
C10B—C11B—H11C	109.5	C21—C22—C23	109.0 (11)
H11A—C11B—H11C	109.5	C21—C22—H22A	109.9
H11B—C11B—H11C	109.5	C23—C22—H22A	109.9
C6A—C5A—C4A	121.5 (11)	C21—C22—H22B	109.9
C6A—C5A—C7A	114.1 (10)	C23—C22—H22B	109.9
C4A—C5A—C7A	124.4 (10)	H22A—C22—H22B	108.3
C10A—C11A—H11D	109.5		
			
C10A—N1A—C8A—C9A	163.9 (10)	O1A—C7A—C5A—C6A	11 (2)
C10A—N1A—C8A—C7A	−83.3 (12)	C8A—C7A—C5A—C6A	−170.2 (13)
O1A—C7A—C8A—N1A	−30.1 (17)	O1A—C7A—C5A—C4A	−169.4 (16)
C5A—C7A—C8A—N1A	151.2 (12)	C8A—C7A—C5A—C4A	9(2)
O1A—C7A—C8A—C9A	84.1 (16)	C4B—C5B—C7B—O1B	172.1 (16)
C5A—C7A—C8A—C9A	−94.6 (14)	C6B—C5B—C7B—O1B	−8(2)
C8B—N1B—C10B—C13B	71.2 (11)	C4B—C5B—C7B—C8B	−8(2)
C8B—N1B—C10B—C11B	−50.9 (13)	C6B—C5B—C7B—C8B	171.9 (13)
C8B—N1B—C10B—C12B	−172.9 (10)	N1B—C8B—C7B—O1B	31.9 (19)
C4B—C5B—C6B—C1B	3(2)	C9B—C8B—C7B—O1B	−94.2 (18)
C7B—C5B—C6B—C1B	−176.9 (16)	N1B—C8B—C7B—C5B	−148.0 (13)
C8A—N1A—C10A—C11A	52.4 (12)	C9B—C8B—C7B—C5B	85.9 (17)
C8A—N1A—C10A—C12A	170.4 (10)	C5B—C6B—C1B—C2B	−6(3)
C8A—N1A—C10A—C13A	−68.4 (11)	C5B—C6B—C1B—Cl1B	176.3 (12)
C10B—N1B—C8B—C7B	81.1 (13)	C3B—C2B—C1B—C6B	6(3)
C10B—N1B—C8B—C9B	−153.4 (11)	C3B—C2B—C1B—Cl1B	−176.5 (12)
C2A—C1A—C6A—C5A	−3(3)	C6A—C1A—C2A—C3A	3(3)
Cl1A—C1A—C6A—C5A	−175.4 (12)	Cl1A—C1A—C2A—C3A	176.0 (14)
C6B—C5B—C4B—C3B	0(2)	C1B—C2B—C3B—C4B	−3(2)
C7B—C5B—C4B—C3B	179.8 (15)	C5B—C4B—C3B—C2B	0(2)
C1A—C6A—C5A—C4A	2(2)	C5A—C4A—C3A—C2A	3(3)
C1A—C6A—C5A—C7A	−178.1 (15)	C1A—C2A—C3A—C4A	−3(3)
C3A—C4A—C5A—C6A	−2(2)	O21—C21—C22—C23	−140.0 (16)
C3A—C4A—C5A—C7A	178.3 (16)		

### Hydrogen-bond geometry (Å, °)

**Table:**

*D*—H···*A*	*D*—H	H···*A*	*D*···*A*	*D*—H···*A*
N1A—H1A1···Br1A	0.90	2.46	3.353 (9)	174.
N1A—H1A2···Br1B^i^	0.90	2.60	3.410 (9)	150.
N1B—H1B1···Br1B^ii^	0.90	2.46	3.362 (9)	175.
N1B—H1B2···Br1A^iii^	0.90	2.58	3.383 (9)	149.
O21—H21···Br1A	0.82	2.73	3.487 (10)	153.

Symmetry codes: (i) *x*−1, *y*, *z*; (ii) *x*, *y*+1, *z*; (iii) *x*+1, *y*+1, *z*.

## References

[bb1] Fang, Q. K., Han, Z., Grover, P., Kessler, D., Senanayake, C. H. & Wald, S. (2000). *Tetrahedron Asymmetry*, **11**, 3659–3663.

[bb2] Farrugia, L. J. (1997). *J. Appl. Cryst.* **30**, 565.

[bb3] Farrugia, L. J. (1999). *J. Appl. Cryst.* **32**, 837–838.

[bb4] Flack, H. D. (1983). *Acta Cryst.* A**39**, 876–881.

[bb5] Froimowitz, M. & George, C. (1998). *J. Chem. Inf. Comput. Sci.* **38**, 506–510. 10.1021/ci980401m 9611786

[bb6] Fryer, J. D. & Lukas, R. J. (1999). *J. Pharmacol. Exp. Ther.* **288**, 88–92. 9862757

[bb7] Higashi, T. (1995). *ABSCOR* Rigaku Corporation, Tokyo, Japan.

[bb8] Maccaroni, E., Malpezzi, L. & Masciocchi, N. (2009). *J. Pharm. Biomed. Anal.* **50**, 257–261. 10.1016/j.jpba.2009.04.021 19464134

[bb9] Rigaku (2006). *PROCESS-AUTO* Rigaku Corporation, Tokyo, Japan.

[bb10] Rigaku (2007). *CrystalStructure* Rigaku, Tokyo, Japan.

[bb11] Sheldrick, G. M. (2008). *Acta Cryst.* A**64**, 112–122. 10.1107/S0108767307043930 18156677

[bb12] Stewart, J. J., Berkel, H. J., Parish, R. C., Simar, M. R., Syed, A., Bocchini, J. A. Jr, Wilson, J. T. & Manno, J. E. (2001). *J. Clin. Pharmacol.* **41**, 770–778. 10.1177/00912700122010564 11452710

